# HIV-1 diversity and pre-treatment drug resistance in the era of integrase inhibitor among newly diagnosed ART-naïve adult patients in Luanda, Angola

**DOI:** 10.1038/s41598-024-66905-1

**Published:** 2024-07-10

**Authors:** Cruz S. Sebastião, Ana B. Abecasis, Domingos Jandondo, Joana M. K. Sebastião, João Vigário, Felícia Comandante, Marta Pingarilho, Bárbara Pocongo, Edson Cassinela, Fátima Gonçalves, Perpétua Gomes, Marta Giovanetti, Ngiambudulu M. Francisco, Euclides Sacomboio, Miguel Brito, Jocelyne Neto de Vasconcelos, Joana Morais, Victor Pimentel

**Affiliations:** 1 Centro de Investigação em Saúde de Angola (CISA), Caxito, Angola; 2 Instituto Nacional de Investigação em Saúde (INIS), Luanda, Angola; 3 Instituto de Ciências da Saúde (ICISA), Universidade Agostinho Neto (UAN), Luanda, Angola; 4 Global Health and Tropical Medicine, GHTM, Associate Laboratory in Translation and Innovation Towards Global Health, LA-REAL, Instituto de Higiene e Medicina Tropical, IHMT, Universidade NOVA de Lisboa, UNL, Rua da Junqueira 100, 1349-008 Lisboa, Portugal; 5 Instituto Nacional de Sangue (INS), Ministério da Saúde, Luanda, Angola; 6 Instituto Nacional de Luta contra SIDA (INLS), Ministério da Saúde, Luanda, Angola; 7 Centro Nacional de Investigação Científica (CNIC), Luanda, Angola; 8 Laboratório de Biologia Molecular (LMCBM, SPC, CHLO-HEM), 1349-019 Lisbon, Portugal; 9 Egas Moniz Center for Interdisciplinary Research (CiiEM), Egas Moniz School of Health & Sicence, Caparica, Almada, Portugal; 10 Department of Science and Technology for Humans and the Environment, University of Campus Bio-Medico di Roma, Rome, Italy; 11 H&TRC-Health & Technology Research Center, ESTeSL-Escola Superior de Tecnologia da Saúde, Instituto Politécnico de Lisboa, Lisbon, Portugal

**Keywords:** HIV-1, Genetic diversity, Drug resistance mutations, INSTI, NGS, Angola, HIV infections, Epidemiology

## Abstract

The surveillance of drug resistance in the HIV-1 naïve population remains critical to optimizing the effectiveness of antiretroviral therapy (ART), mainly in the era of integrase strand transfer inhibitor (INSTI) regimens. Currently, there is no data regarding resistance to INSTI in Angola since Dolutegravir-DTG was included in the first-line ART regimen. Herein, we investigated the HIV-1 genetic diversity and pretreatment drug resistance (PDR) profile against nucleoside/tide reverse transcriptase inhibitors (NRTIs), non-nucleoside reverse transcriptase inhibitors (NNRTIs), protease inhibitors (PIs), and INSTIs, using a next-generation sequencing (NGS) approach with MinION, established to track and survey DRMs in Angola. This was a cross-sectional study comprising 48 newly HIV-diagnosed patients from Luanda, Angola, screened between March 2022 and May 2023. PR, RT, and IN fragments were sequenced for drug resistance and molecular transmission cluster analysis. A total of 45 out of the 48 plasma samples were successfully sequenced. Of these, 10/45 (22.2%) presented PDR to PIs/NRTIs/NNRTIs. Major mutations for NRTIs (2.2%), NNRTIs (20%), PIs (2.2%), and accessory mutations against INSTIs (13.3%) were detected. No major mutations against INSTIs were detected. M41L (2%) and I85V (2%) mutations were detected for NRTI and PI, respectively. K103N (7%), Y181C (7%), and K101E (7%) mutations were frequently observed in NNRTI. The L74M (9%) accessory mutation was frequently observed in the INSTI class. HIV-1 pure subtypes C (33%), F1 (17%), G (15%), A1 (10%), H (6%), and D (4%), CRF01_AG (4%) were observed, while about 10% were recombinant strains. About 31% of detected HIV-1C sequences were in clusters, suggesting small-scale local transmission chains. No major mutations against integrase inhibitors were detected, supporting the continued use of INSTI in the country. Further studies assessing the HIV-1 epidemiology in the era of INSTI-based ART regimens are needed in Angola.

## Introduction

The rapid mutation rate inherent to HIV-1 replication plays a crucial role in its widespread transmission. This characteristic gives the virus the ability to evade host immune responses and antiretroviral treatment (ART) and it also contributes significantly to genetic diversity^1^. The emergence of drug resistance mutations (DRMs) poses a threat to the effectiveness of ART, mainly in low- and middle-income countries (LMICs)^2^. The WHO recommends implementing strategies to prevent HIV drug resistance (HIVDR), aiming to sustain the progress achieved through the scale-up of ART, improving patient well-being and reducing associated program costs^3^. These measures include monitoring the occurrence of pretreatment drug resistance (PDR) among individuals initiating ART^3^.

Currently, HIV-1 subtype C (HIV-1C), constitutes approximately 47% of global infections, followed by subtypes B (12%) and A (10%)^4^. In Angola, approximately 310,000 people are living with HIV^5^. The last study on HIV-1 molecular epidemiology conducted in 2018 has shown a high genetic diversity with HIV-1C being the most common subtype in Angola, accounting for approximately 38% of infections, followed by subtypes F1 (18%), G (9%), A1 (9%), D (6%) and H (3%)^6^. In response to the worldwide rise in drug resistance to non-nucleoside reverse transcriptase inhibitors (NNRTIs), WHO has advised the shift from NNRTI to integrase strand transfer inhibitor (INSTI)-based treatment plans for both individuals who have or have not received prior treatment^7^. Numerous LMICs including Angola, have already adopted the dolutegravir (DTG) based treatment in combination with two nucleoside reverse transcriptase inhibitors (NRTIs)^8^.

To date, Sanger sequencing capable of identifying mutations with a prevalence of 15–25% or more has been used to analyse specific viral protein-coding sequences encoding ARVs drug targets^9^. In contrast, deep sequencing approaches based on nanopore technology (Oxford Nanopore Technologies [ONT]), have been developed for DR testing as they are capable of detecting less abundant mutations (< 15%), although the clinical impact of detecting such Low-abundance mutations remain controversial and merit further investigation^10^. The next-generation sequencing (NGS) approach based on ONT technology is cost-effective, portable/accessible and applied in genome assembly, full-length transcript detection and base modification detection and in more specialised areas, such as rapid clinical diagnoses and outbreak surveillance for countries where resources are limited, but the burden of infectious diseases such as the HIV is high^11^.

Currently, HIV genotyped data is scarce in Angola and there is no published data regarding drug resistance to INSTIs since DTG was included in the first-line ART regimen in 2021. In the present study, we investigated for the first time, the most recent epidemiological profile of HIV-1 genetic diversity and PDR profile against NRTIs, NNRTIs, PIs, and INSTIs, using an NGS approach established to track and survey DRMs in Angola. Our findings will inform the clinical advantages of adopting baseline resistance testing for integrase inhibitors using the NGS approach in Angola^12^.

## Materials and methods

### Study design and setting

This was a cross-sectional study based in a health unit, the National Blood Institute, a healthcare unit specializing in the screening and treatment of infectious diseases, located in Luanda, the capital city of Angola. The study was conducted with 48 adult individuals male or female recently diagnosed with HIV and who had not been exposed to ART. The initial diagnosis of all patients was between March 2022 and May 2023. All participants were reactive for anti-HIV (Abbott, USA) using the ARCHITECT Plus i2000SR Immunoassay Analyzer (Abbott, USA). At the time of recruitment, the study participants were asymptomatic and older than eighteen. The study was conducted in compliance with the Declaration of Helsinki. The study protocol was reviewed and approved by the National Ethics Committee of the Angolan Ministry of Health (MoH) (protocol number 39/C.E./2021, dated December 1st, 2021) and by the National Blood Transfusion Service in Angola (protocol number 128/GDG/INS/2022, dated February 24th, 2022). All participants provided their verbal informed consent before being included in the study.

### Data collection and laboratory procedure

A structured epidemiological questionnaire was drawn up and applied during the inclusion of the participants to collect sociodemographic data such as age, sex, area of residence, and occupation. All participant data were anonymized and used only for the proposed study. In addition, around 5 mL of whole blood sample was collected intravenously from each participant and stored in tubes containing ethylenediaminetetraacetic acid (EDTA). The whole blood plasma samples were then separated by centrifugation and the plasma was frozen at − 80 °C.

### RNA extraction, PCR amplification, sequencing, and genome assembly

Total viral RNA was extracted from 140 µL (µL) of thawed plasma samples from the participants using the QIAamp Viral RNA kit (QIAGEN, Germany) following the manufacturer's instructions. For sequencing purposes, amino acid positions 1–99 in the protease (PR) (HXB2 position: 2253–2549), 1–238 in the reverse transcriptase (RT) (HXB2 2550–3263) and 1–267 in the integrase (INT) (HXB2 4230–5030) were amplified from 5 µL of extracted RNA in a 20 µL final PCR reaction mixture containing Taq Platinum High Fidelity enzyme and primers. Two one-step PCRs were conducted independently for each plasma sample followed by a nested PCR, using an in-house protocol with the primer sets previously published^13^. The final PCR products were revealed through electrophoresis using 1% of the agarose gel, whether PR/RT (1112 bp) or IN (1254 bp) fragment was present. Amplicons were purified using 1xAMPure XP beads (Beckman Coulter, Brea, CA, USA), and all the sample concentrations were normalized to an initial input of 80 ng/µL for each sample including PR, RT, and IN fragments. DNA library preparation was conducted using the Ligation Sequencing kit (SQK-NBD114.24, Oxford Nanopore Technologies) and Native Barcoding Expansion 1–24 kit (R10.4.1, Oxford Nanopore Technologies), following the reaction conditions as previously described^14^. Sequencing was performed for up to 12 h on a MinION Mk1C Sequencing Device. Reads were mapped and aligned against sample-specific reference sequences constructed for the *pol*-PR/RT/IN genomic region using the Geneious Prime v2024.0.5 (https://www.geneious.com/features/prime) and the assembled FASTQ files exported. In the context of this research, we considered the FASTA file generated by the assay that corresponds to one consensus nucleotide sequence per isolate. In this sequence, codons that contained mixtures of nucleotides present in variants that corresponded to or exceeded 5% of the viral populations were denoted using the International Union of Pure and Applied Chemistry (IUPAC) ambiguity code^15–17^. The consensus FASTA file sequences were exported to HIV-1 subtyping and drug resistance analysis.

### HIV‑1 subtyping determination and transmitted drug resistance analysis

The FASTA files generated were submitted to REGA HIV-1 Subtyping Tool version 3.46^18^ and Comet genotyping tools^19^ to examine the HIV-1 genetic diversity. Therefore, the nucleotide sequences were submitted to the Calibrated Population Resistance (CPR) analysis tool (https://hivdb.stanford.edu/cpr/) to calculate the proportions of individuals with overall NRTI, NNRTI, PI, and INSTI‐associated PDR taking into account the list published by Tzou et al., 2020^20^. The clinical impact of genotypic drug resistance on first-line ART was assessed using HIVdb Program version 9.1.0, https://hivdb.stanford.edu/hivdb/by-patterns/.

### Phylogenetic analysis of the Angolan HIV-1C sequences

A maximum-likelihood (ML) phylogenetic tree of the HIV-1C sequences from drug-naïve HIV-infected individuals detected in this study was constructed with other 129 HIV-1C control sequences (https://www.hiv.lanl.gov/content/sequence/BASIC_BLAST/basic_blast.html). The sequences were aligned with Viruglin^21^ and the ML tree was constructed using the FastTree^22^ with the GTR parameter as the substitution model.

### Statistical analysis

The statistical analysis was performed in SPSS v29 (IBM SPSS Statistics, USA). The descriptive analysis was presented as absolute (N) and relative (%) frequencies. The normal data distribution was checked with the Shapiro–Wilk test and compared with the independent-sample T-test. Mean and standard deviation (SD) were presented to normal distribution data. Qualitative variables such as age (≤ 30 years old and > 30 years old), sex (female and male), residence area (non-urbanized and urbanized), and occupation (unemployed and employed) were dichotomized, and proportions analysed with the Chi-square (X2) or Fisher's exact test, as appropriate, to predict demographic characteristics related to PDR. All reported *p*-values are two‐tailed and were considered statistically significant when p < 0.05.

### Ethics declarations

The study was conducted following the Declaration of Helsinki and approved by the Ethics Committee of the Ministry of Health of Angola (protocol number 39/C.E./2021, approved on December 1st, 2021) and by the National Blood Transfusion Service in Angola (protocol number 128/GDG/INS/2022, approved on February 24th, 2022). Also, informed consent was obtained from all subjects involved in the study.

## Results

### Demographic characteristics related to drug resistance mutations

**Figure 1:**
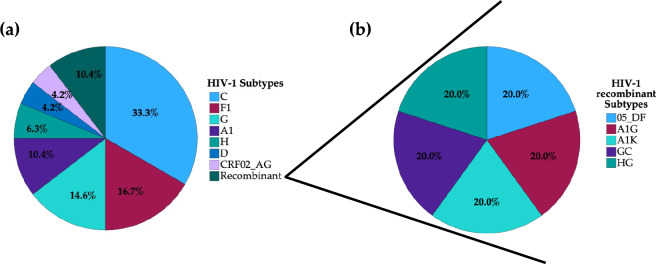
HIV-1 genetic diversity. (**a**) Overall HIV-1 subtypes. (**b**) Other HIV-1 strains. REGA version 3.46 HIV-1 Subtyping Tool and Comet genotyping tools were used to examine the HIV-1 genetic diversity.

The demographic characterization of the participants and the putative features related to PDR are presented in Table 1. A total of 48 newly diagnosed HIV-1 patients from Luanda, the capital city of Angola, were enrolled in the present study. The age of the participants ranged from 19 to 57 years old, with a mean of 34.4 ± 8.84 years old. Patients aged over 30 years (56.3%, 27/48), male (87.5%, 42/48), living in non-urbanized areas (52.1%, 25/48), and with any type of occupation whether in the public or private sector (81.3%, 39/48), were the most predominant. A total of 48 plasma samples were successfully sequenced for PR/RT and IN. It was not possible to perform any quantification of CD4 cells and the viral load was quantified in only 29 of the 48 included in the study. The viral load varied from 1.64 to 5.69 Log_10_, with a mean of 4.50 ± 1.10 Log_10_. No drug resistance was observed to integrase inhibitor. The prevalence of PDR in the PR/RT was 22.2% (10/45). None of the demographic characteristics studied (e.g. age groups, sex, place of residence, and occupation) were statistically related to PDR (p > 0.05). Patients with PDR (32.6 ± 10.2 years old) had a lower average age compared to patients without PDR (35.3 ± 8.39 years old), with a difference of 2.71 years in age. Regarding the genetic variability of the HIV-1 strains infecting those patients, subtype C (33.3%, 16/48) was the most predominant, followed by subtypes F1 (16.7%, 8/48), G (14.6%, 7/48), A1 (10.4%, 5/48), H (6.3%, 3/48), D (4.2%, 2/48), and CRF02_AG (4.2%, 2/48). Recombinant strains represented 10.4% (5/48) (Fig. 1a). The recombinant strains such as CRF05_DF, A1G, A1K, GC, and HG represented about 20% each within the category of other HIV-1 strains (Fig. 1b).

**Table 1:** Demographic description and features related to PDR among HIV-1 newly diagnosed in Angola.

Independent variable	N (%)	Any PDR to PR/RT/IN^§^		
		No (%)	Yes (%)	p-value^#^
Overall	48 (100)	35 (77.8)	10 (22.2)	
Age (year)—mean ± SD	34.4 ± 8.84	35.3 ± 8.39	32.6 ± 10.2	0.394
Age distribution				
≤ 30 years	21 (43.8)	13 (68.4)	6 (31.6)	0.281
> 30 years	27 (56.3)	22 (84.6)	4 (15.4)	
Sex				
Female	6 (12.5)	4 (80.0)	1 (20.0)	0.899
Male	42 (87.5)	31 (77.5)	9 (22.5)	
Residence area				
Non-urbanized	25 (52.1)	18 (78.3)	5 (21.7)	0.936
Urbanized	23 (47.9)	17 (77.3)	5 (22.7)	
Occupation				
Unemployed	9 (18.8)	5 (62.5)	3 (37.5)	0.349
Employed	39 (81.3)	30 (81.1)	7 (18.9)	
HIV-1 subtypes				
C	16 (33.3)	11 (68.8)	5 (31.3)	0.460
F1	8 (16.7)	4 (57.1)	3 (42.9)	
G	7 (14.6)	5 (100)	0 (0.0)	
A1	5 (10.4)	5 (100)	0 (0.0)	
H	3 (6.30)	2 (66.7)	1 (33.3)	
D	2 (4.20)	2 (100)	0 (0.0)	
CRF02_AG	2 (4.20)	2 (100)	0 (0.0)	
Other HIV-1 strains	5 (10.4)	5 (100)	0 (0.0)	

^#^Chi-square (X^2^) test or Fisher's exact test.

^§^Three samples did not amplify for the three PR/RT/IN fragments, simultaneously.

### Distribution of pretreatment drug resistance according to ARV classes and patient demographic characteristics

**Figure 2:**
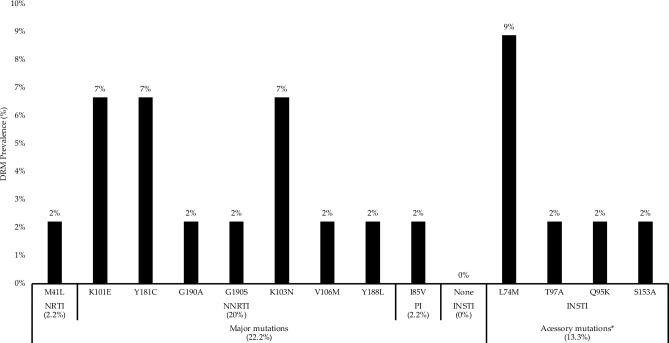
Prevalence of drug resistance mutations according to antiretroviral classes. *NRTI* nucleoside reverse transcriptase inhibitors, *NNRTI* non-nucleoside reverse transcriptase inhibitors, *PI* protease inhibitors, *INSTI* Integrase Strand Transfer Inhibitor. Calibrated Population Resistance (CPR) tool version 8.1 and the HIVdb program, which are available at Stanford University HIV Drug Resistance Database (HIVdb) (https://hivdb.stanford.edu/) were used to assess DRMs according to the ARV classes. *No major mutations were observed to integrase inhibitors.

Table 2 presents the distribution of PDR according to ARV drug classes to PR/RT and the patient's demographic characteristics. The major mutations were detected in NRTIs (2.2%), NNRTIs (20%), and PIs (2.2%). Occupation was statistically related to the presence of PDR for NRTIs (p = 0.030). No major mutations were detected in INSTIs, although 13.3% of the studied population presented accessory mutations (L74M, T97A, Q95K, and S153A) to INSTIs. The K103N (7%), Y181C (7%), and K101E (7%) mutations were frequently observed in NNRTI. The M41L (2%) mutation against NRTI and I85V (2%) mutation against PI were detected. The L74M (8%) accessory mutation was the most frequent in the INSTI drug class, followed by the T97A, Q95Q, and S153A (Fig. 2). As shown in Table 3, only of the patients (ID: HIT166; PI: I85V and NNRTI: K103N) showed resistance to multiple drug classes. On the other hand, multiple mutations of the same class were observed in two patients [(ID: HIT919; NNRTI: K101E, Y181, G190A) and (ID: HIT1218; NNRTI: K101E, Y181C, G190S)]. The I85V mutation in PI was detected in 86% of 29,235 reads; The M41L mutation in NRTI was detected in 88% of 1938 reads; The Y188L mutation in NNRTI was detected in 86% of 30,907 reads; The K101E mutation in NNRTI was detected in 35–48% of approximately 9060–27,180 reads; The K103N mutation in NNRTI was detected between 87 and 89% in approximately 7434–56,083 reads; The Y181C mutation in NNRTI was detected between 88 and 89% in approximately 1812–25,956 reads; The G190AS mutation in NNRTI was detected between 91 and 94% in approximately 8895–25,922 reads; the Y188L mutation in NNRTI was detected in 85% of 11,805 reads (Table 3).

**Table 2:** Distribution of transmitted drug resistance according to ARV classes and demographic characteristics of the Angolan naïve patients.

Independent variable	N (%)	NRTI mutation			NNRTI mutation			PI mutation		
		No (%)	Yes (%)	p-value	No (%)	Yes (%)	p-value	No (%)	Yes (%)	p-value
Overall	45 (100)	44 (97.8)	1 (2.2)		36 (80.0)	9 (20.0)		44 (97.8)	1 (2.2)	
Age distribution										
≤ 30 years	19 (42.2)	19 (100)	0 (0.0)	1.000	13 (68.4)	6 (31.6)	0.137	18 (94.7)	1 (5.3)	0.422
> 30 years	26 (57.8)	25 (96.2)	1 (3.8)		23 (88.5)	3 (11.5)		26 (100)	0 (0.0)	
Sex										
Female	5 (11.1)	5 (100)	0 (0.0)	1.000	4 (80.0)	1 (20.0)	1.000	5 (100)	0 (0.0)	1.000
Male	40 (88.9)	39 (97.5)	1 (2.5)		32 (80.0)	8 (20.0)		39 (97.5)	1 (2.5)	
Residence área										
Non-urbanized	23 (51.1)	23 (100)	0 (0.0)	0.489	18 (78.3)	5 (21.7)	0.766	23 (100)	0 (0.0)	0.489
Urbanized	22 (48.9)	21 (95.5)	1 (4.5)		18 (81.8)	4 (18.2)		21 (95.5)	1 (4.5)	
Occupation										
Unemployed	8 (17.8)	7 (87.5)	1 (12.5)	**0.030**	6 (75.0)	2 (25.0)	0.651	8 (100)	0 (0.0)	0.638
Employed	37 (82.2)	37 (100)	0 (0.0)		30 (81.1)	7 (18.9)		36 (97.3)	1 (2.7)	
HIV-1 subtypes										
C	16 (35.6)	16 (100)	0 (0.0)	0.593	11 (68.8)	5 (31.3)	0.609	15 (93.8)	1 (6.3)	0.967
F1	7 (15.6)	6 (85.7)	1 (14.3)		5 (71.4)	2 (28.6)		7 (100)	0 (0.0)	
G	5 (11.1)	5 (100)	0 (0.0)		5 (100)	0 (0.0)		5 (100)	0 (0.0)	
A1	5 (11.1)	5 (100)	0 (0.0)		4 (80.0)	1 (20.0)		5 (100)	0 (0.0)	
H	3 (6.7)	3 (100)	0 (0.0)		2 (66.7)	1 (33.3)		3 (100)	0 (0.0)	
D	2 (4.4)	2 (100)	0 (0.0)		2 (100)	0 (0.0)		2 (100)	0 (0.0)	
CRF02_AG	2 (4.4)	1 (100)	0 (0.0)		2 (100)	0 (0.0)		2 (100)	0 (0.0)	
Other HIV-1 strains	5 (11.1)	5 (100)	0 (0.0)		5 (100)	0 (0.0)		5 (100)	0 (0.0)	

Bold number indicate statistical significance for the Chi-square test or Fisher's exact test (p < 0.05).

**Table 3:** Distribution of pretreatment drug resistance according to PI, NRTI, NNRTI and INSTI dug classes and HIV-1 subtypes.

ID_project	HIV-1 subtypes	Pretreatment drug resistance			
		PI	NRTI	NNRTI	INST
HIT21	F1	None	None	Y188L (86%; cov = 30.907)	None
HIT127	C	None	None	K101E (35%; cov = 17.019)	None
HIT166	C	I85V (86%; cov = 29.235)	None	K103N (87%; cov = 29.208)	None
HIT225	C	None	None	Y181C (88%; cov = 1.812)	None
HIT919	A1	None	None	K101E (45%; cov = 9.060) Y181C (89%; cov = 8.900) G190A (94%; cov = 8.895)	None
HIT1002	H	None	None	K103N (88%; cov = 56.083)	None
HIT1218	C	None	None	K101E (48%; cov = 27.180) Y181C (88%; cov = 25.956) G190S (91%; cov = 25.922)	None
HIT1281	C	None	None	V106M (85%; cov = 11.805)	None
HIT1523	F1	None	M41L (88%; cov = 1.938)	None	None
HIT1808	F1	None	None	K103N (89%; cov = 7.434)	None

### Phylogenetic tree of the HIV-1C sequences

**Figure 3:**
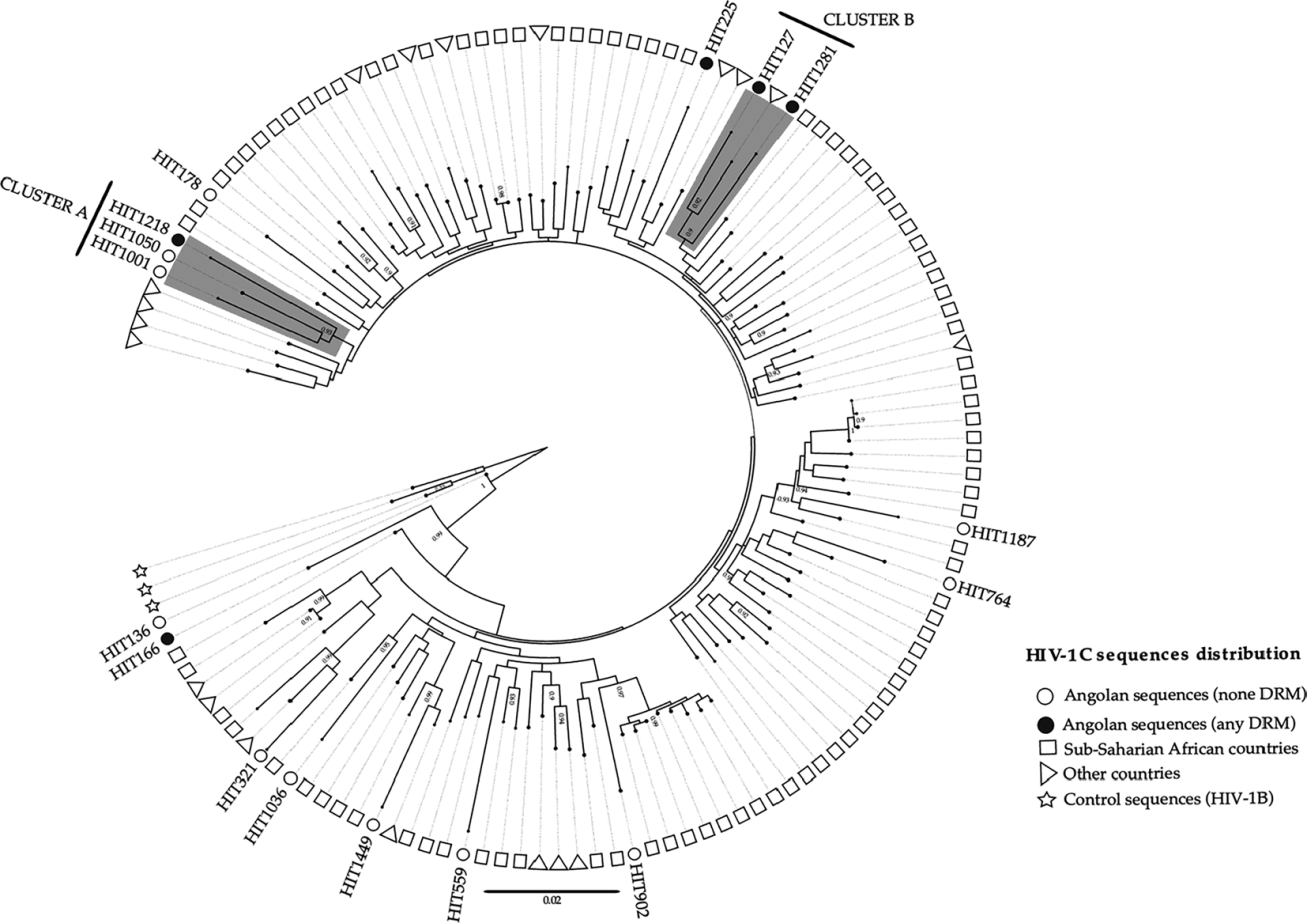
Maximum-Likelihood (ML) phylogenetic tree of the HIV-1C sequences from the 16 ART-naïve HIV-infected individuals. Sequences were aligned with the Virulign and the phylogenetic tree constructed using FastTree. Bootstrapping was performed with 1000 replicates and only those that had a minimum bootstrap value of 90 were indicated on the tree. Additional HIV-1C sequences were retrieved from the HIV-1 Los Alamos National Lab (LANL) HIV Sequence Database (https://www.hiv.lanl.gov/content/sequence/BASIC_BLAST/basic_blast.html). Also, three HIV1B retrieved from the HIV-1 LANL HIV Sequence Database sequences represented as stars were included as control sequences. All HIV-1C Angolan sequences from this study are indicated as white circles (if no DRM is observed) and dark circles (if any DRM is observed). Sequences from sub-Saharan African countries were represented as squares and a triangle was used to represent sequences from other countries in America, Europe, and Asia.

The HIV-1C sequences detected in this study population (33.3%, 16/48) were mostly related to HIV-1C sequences from Sub-Saharan African countries (e.g., HIV-1C sequences from South Africa, Botswana, and Zambia) and America (e.g. Brazil and the United States). A total of 5/16 of the HIV-1C Angolan sequences detected in the present study (31.3%) showed two clusters (Cluster A and Cluster B) suggestive of transmission cluster with monophyletic groups (≥ 90% of branch support SH-aLRT) (Fig. 3). Cluster A represents three of our samples, one of which (HIT1218) had three NNRTI-associated resistance mutations (K101E, Y181C, and G190S). Cluster B is composed of three sequences, two related to our study that share mutations associated with the same class, the NNRTI, as also shown in Table 3.

## Discussion

Due to an increase in resistance rates against NNRTIs^23^, INSTI drug classes such as dolutegravir-DTG, which exhibit an elevated genetic barrier to resistance are being used in the first line of ART regimens^24^. In Angola, where there are no established procedures for routine HIV-1 genotyping or regular monitoring of DRMs, the adoption of DTG took place around 2021, aligning with WHO guidelines^25^. To our knowledge, this is the most recent study that provides an update on the prevalence of PDR against NNRTIs, NRTIs, and, PIs among untreated HIV-1 infected people in Angola, after 5 years of a previous study conducted by our research team^6^. Also, this is the first study that presents drug resistance data to INSTIs in Angola.

We did not detect any mutation of clinical interest against INSTI, which supports the continued use of INSTI in the country. None or low resistance levels to INSTIs were also observed in previous studies from sub-Saharan African regions, such as East African countries (0%)^26^, Ethiopia (0%)^27^, Nigeria (0%)^28^, Cameroon (1.4%)^29^, and South Africa (2.2%)^30^, as well as in America such as in Brazil (0%)^31^ and the US (1.9%)^32^, or 2.3% in Europe as reported in the MeditRes which is a consortium that includes ART-naïve patients newly diagnosed in France, Greece, Italy, Portugal, and Spain during 2018–2021^33^. On the other hand, we detected accessory mutations (L74M, T97A, Q95K, and S153A) that have little effect unless they present with other major mutations (Fig. 2). The overall prevalence of accessory mutations in the present study was 13.3%, which is higher than the detection rate reported in the sub-Saharan African region (8.7%)^23^. Among the accessory mutations detected, L74M was the most common, which is in line with previous studies carried out in sub-Saharan Africa showing that L74M and T97A are the most common INSTI accessory mutations in this region^23^. L74M is a polymorphic mutation selected by all INSTI drugs and occurs at varying degrees (0.5–20%) in ART-naïve populations infected with subtypes A and G^34^. It is worth mentioning that all the studied patients who presented accessory mutations for INSTI had non-HIV-1C subtypes (data not shown), while 50% (5/10) of the sequences with mutations for PI, NRTI and NNRTI were subtype C (Table 3). Among this non-HIV-1C, subtype A1 with 50% and subtypes F1, G, and H, simultaneously with 16.7%, were predominant, showing that although HIV-1C are largely driving the epidemic in Angola as described by previous studies^6,35,36^, drug resistance to the INSTI class could be driven by non-HIV-1C subtypes. Indeed, similar reports showed that L74M and T97A accessory mutations were more frequent in patients infected with non-HIV-1C subtypes, such as, subtypes A, G, and recombinant strains, which is in line with our findings^37^. L74M alone has minimal if any, effect on INSTI susceptibility, however, combined with major INSTI mutations, mainly G140 and Q148, it significantly contributes to reduced susceptibility to each of the INSTIs drugs^34,38^. Then, this detected accessory mutation in high prevalence among the Angolan ART-naïve patients reinforces the need to intensify surveillance of polymorphisms associated with ARV resistance.

The prevalence of PDR for PR/RT observed in this study (22%) was higher than that reported in several East African countries (10%)^26^, which reinforces the current WHO recommendation to replace NNRTIs (which had a 20% prevalence in the present study, Fig. 2) with INSTIs in ART regimens^25^. On the other hand, the PDR against NRTIs and PIs although low, was notable and corresponds with the findings observed in other regions of Africa^39^, observed in the PharmAccess African Studies to Evaluate Resistance Monitoring among ART-naive individuals which reported 2.5% for NRTIs, 3.3% for NNRTIs, 1.3% for PIs and 1.2% for dual-class resistance to NRTIs and NNRTIs^40^, which justifies continued surveillance of DRMs against NRTIs administered alongside DTG in Angola. For the NRTI drug class, we detected the M41L mutation associated with reduced susceptibility to zidovudine-AZT, abacavir-ABC, and tenofovir-TDF^35^. For the NNRTI class, we detected the K103N, K101E, and Y181C mutations, known to confer high resistance to NVP or EFV, mainly the K103N mutations which pose an important threat to the clinical ART response in patients undergoing treatment with regimens containing EFV^41^. The I85V mutation was the only one detected in PIs, and this mutation has minimal if any, effects on PI susceptibility, indicating that PIs continue to be effective and are still a good choice for treating patients with HIV-1 in Angola^42^.

Our phylogenetic analysis showed that the HIV-1C lineage that circulates in Luanda, the capital city of Angola, has a genetic signature corresponding to the lineages that circulate predominantly in other sub-Saharan African countries, notably South Africa, Botswana, and Zambia where subtype C also predominates^43^. About 30% of the HIV-1C sequences in the present study were in clusters, which could be indicative of small-scale local transmission chains (comprising 2 and 3 sequences) (Fig. 3). Interestingly, we observed the clustering of Angolan sequences with Brazilian and European counterparts, plausibly explicable by historical and economic ties between Angola, Brazil, and Portugal. These findings highlighted that international migration across the country's borders is a significant driving force for recurrent introductions of HIV-1C strains as well as multiple HIV-1 subtypes, which continue to maintain the Angolan HIV-1 epidemic profile characterised by high genetic diversity^6,35,36,44^. It is worth mentioning that we detected Angolan HIV-1C sequences carrying drug mutations to NNRTIs such as efavirenz-EFV and nevirapine-NVP, commonly used in the ART regimen in Angola, which belonged to the same monophyletic cluster with a branch support of 90% (Fig. 3). This finding highlights the potential dissemination of resistant virus strains in the general population, showing the importance of conducting studies combining phylogenetic, clinical, and epidemiological data as an effective strategy to identify possible key populations or transmission clusters that driven resistant HIV strains among PLWH in Angola.

This study has important limitations. The limited number of enrolled patients may not be sufficient to represent the current picture of the molecular epidemiology of HIV in ART-naïve patients from Luanda or the whole infected population of Angola, reinforcing the need to conduct other studies with a larger population and from different regions. We also recognise that more appropriate phylogenetic analyses with Bayesian models would be essential for a better understanding of the possible origins and introduction dates of a specific lineage, but the phylogenetic tree presented in this study was fundamental to identifying the putative relationships and migratory flow between Angolan and sub-Saharan African HIV-patients^45^. Despite these limitations, our findings shed light on the current scenario of molecular epidemiology and drug resistance patterns in ART-naïve HIV patients in Angola. Moreover, the study showed that monitoring patients who begin treatment with DTG and applying the NGS approach is crucial to obtaining data on ART response and drug resistance patterns highlighting Angola as one of the key countries in the fight against the HIV-1 pandemic in Africa.

## Conclusions

In summary, we did not detect any major INSTI mutation associated with dolutegravir-DTG resistance, suggesting that ART regimens containing DTG will be effective among the ART-naïve patients from Angola, which supports the wide use of INSTI drugs as part of the first-line ART regimens. HIV-1 subtype C remains to drive the HIV epidemic in Angola. A small-scale local transmission cluster of HIV-1C strains has been identified, warranting additional attention from public health authorities. It is crucial to direct efforts towards monitoring the response and drug resistance patterns to ART in patients undergoing DTG treatment in Angola. Further studies using NGS approaches will be crucial to track and survey the molecular epidemiology of HIV-1 as well as monitoring and addressing drug resistance in the era of INSTI-based ART regimens in Angola.

## Data Availability

The datasets generated during and/or analysed during the current study are available from the corresponding author upon reasonable request. Moreover, the sequences obtained in the study were deposited to GenBank (NCBI) and were assigned the accession numbers PP776478 to PP776525.
